# Native Valve Endocarditis due to *Ralstonia pickettii*: A Case Report and Literature Review

**DOI:** 10.1155/2015/324675

**Published:** 2015-01-11

**Authors:** Joseph Orme, Tomas Rivera-Bonilla, Akil Loli, Negin N. Blattman

**Affiliations:** ^1^Department of Internal Medicine, Banner Good Samaritan Medical Center, Phoenix, AZ 85006, USA; ^2^Department of Cardiology, Banner Good Samaritan Medical Center, Phoenix, AZ 85006, USA; ^3^Biltmore Cardiology, Phoenix, AZ 85018, USA; ^4^Phoenix VA Healthcare System, 650 E Indian School Road, Phoenix, AZ 85012, USA

## Abstract

*Ralstonia pickettii* is a rare pathogen and even more rare in healthy individuals. Here we report a case of *R. pickettii* bacteremia leading to aortic valve abscess and complete heart block. To our knowledge this is the first case report of *Ralstonia* species causing infective endocarditis with perivalvular abscess.

## 1. Case Report

**Figure 1:**
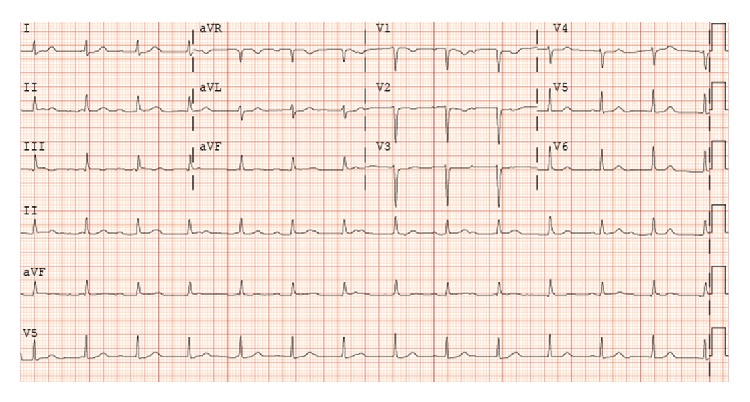
ECG demonstrating complete atrioventricular block with accelerated junctional escape.

A 51-year-old female with a past medical history of deep vein thrombosis (DVT), pulmonary embolism, and well controlled diabetes mellitus type 2 (hemoglobin A1c 6.1%) presented after several days of worsening chest pain, low-grade fevers, and chills. Several weeks prior to presentation patient had a central venous catheter placed for intravenous iron infusions to treat refractory iron-deficiency anemia. Three weeks prior to presentation, the patient had left tarsal tunnel release with no postoperative complications. Upon presentation, to the hospital for evaluation she was bradycardic with a pulse of 48 beats per minute, hypotensive with a blood pressure of 106/54 mmHg (as compared to her baseline hypertension), and febrile to 101.3°C. Given her history of DVT, a computed tomography (CT) angiogram was ordered that revealed no new pulmonary emboli but showed cavitary lung lesions suggestive of septic emboli. An electrocardiogram (ECG) demonstrated accelerated junctional escape rhythm with complete atrioventricular block (Figure 1). Blood and urine cultures were obtained, and patient was initiated on empiric coverage for endocarditis with vancomycin, gentamicin, and micafungin.

**Figure 2:**
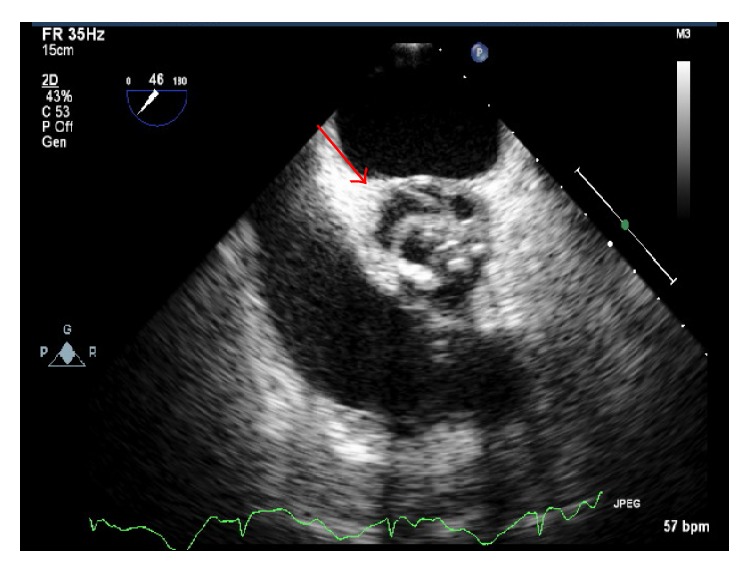
Transesophageal echocardiogram at midesophageal inflow/outflow tract revealing subannular abscess, in diastole.

**Figure 3:**
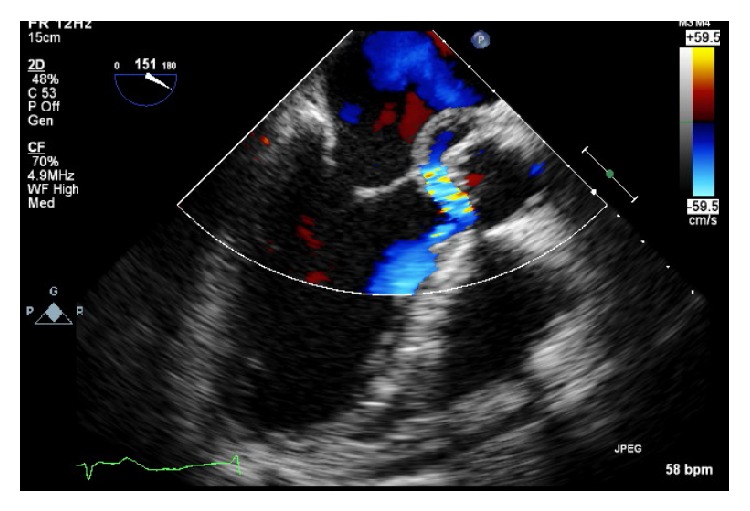
Transesophageal echocardiogram at midesophageal long-axis view with Doppler revealing regurgitation into abscess surrounding the aortic valve suggestive of aortic fistula.

Given CT evidence of septic emboli, fevers, and ECG findings of complete AV block, an initial transthoracic echocardiogram (TTE) was performed on day two of admission, followed by a transesophageal echocardiogram (TEE) on day three of admission. TEE confirmed initial TTE findings of aortic valve thickening on the left coronary cusp highly suggestive of vegetation (Figure 2) and associated severe aortic regurgitation. Furthermore, an echo density was noted at the aortic root with color flow transmission highly suggestive of an aortic root abscess with fistula (Figure 3). There was moderate mitral valve regurgitation with normal left ventricular systolic function. A bicuspid aortic valve was also noted on the TEE. Gram-positive cocci were seen on Gram stain from blood cultures drawn on admission; therefore she was continued on vancomycin and gentamicin. The patient was referred for emergent cardiothoracic surgery with replacement of the aortic valve with a 19 mm freestyle tissue valve, incision and drainage and debridement of the subannular abscess, and reconstruction of the proximal anterior leaflet of the mitral valve and aortic annulus with pericardial patch placement which was performed at an outside hospital on day six of hospitalization. No pacemaker was placed at this time of surgery as the cardiothoracic surgeons felt that it would best be placed once her blood cultures were sterile. At the time of valve replacement a transfemoral pacer was placed.

Within 24 hours of hospitalization, blood cultures drawn on admission began growing what was initially identified as Gram-positive cocci. However, on day three of admission the Gram stain was reassessed and changed to Gram-negative rods identified as* Ralstonia* species. Repeat blood cultures on consecutive days up until the day of surgery grew persistent* Ralstonia* species, which was ultimately identified as* Ralstonia pickettii*. Surgical specimens from the aortic valve and annular abscess all had heavy growth of* R. pickettii* (surgical intervention on day 6). All postsurgical blood cultures remained negative (Table 1). She was initially on aggressive Gram-positive coverage initially with vancomycin and gentamicin; however this was quickly changed to levofloxacin once sensitivities returned. The* Ralstonia* species, later identified as* pickettii*, was sensitive to quinolones and trimethoprim-sulfamethoxazole only with intermediate sensitivity to piperacillin/tazobactam, imipenem, and cefepime and complete resistance to tobramycin amikacin and gentamycin. Her postoperative course was uneventful except for dental extractions done for extensive necrosis and caries. She was initiated on levofloxacin on day four of admission and completed a total of eight weeks of therapy postoperatively. Upon sterilization of blood cultures approximately one week after surgery, a dual-chamber pacemaker was implanted.

Unfortunately, shortly after completion of the initial eight weeks of antibiotic therapy, the patient developed recurrent bacteremia with* Ralstonia pickettii* complicated by a periannular abscess around the new aortic valve prosthesis and a pseudoaneurysm of the ascending aorta. She was again emergently taken for repeat aortic root replacement with a 24 mm homograft and treated with aggressive antibiotic therapy with trimethoprim-sulfamethoxazole and levofloxacin (after repeat sensitivity testing). Unfortunately the patient rapidly succumbed to infection and died due to complications of persistent bacteremia.

*Ralstonia* species are aerobic Gram-negative, oxidase-positive, nonfermenting bacilli that have in recent years been identified as emerging opportunistic pathogens in immunocompromised hosts. Both environmental and hospital sources have been identified in human infection. Of the* Ralstonia* genus,* Ralstonia pickettii* formerly known as* Burkholderia pickettii* is regarded as the one with clinical importance [1]. It was first identified as* Pseudomonas pickettii* in 1973 [2] and then reclassified in 1992 to the* Burkholderia* [3] genus and finally in 1995 to a new genus* Ralstonia* [4–6], based upon cellular lipid and fatty acid composition, phenotypic analysis, and both DNA and 16s rRNA sequencing and hybridization. Disease associated with* Ralstonia pickettii* ranges from asymptomatic to septicemia and death.

## 2. Discussion

Historically, the first documented case of* Ralstonia* bacteremia and death was reported in 1968 [7]. At that time, the pathogen was reported as an unclassified, Gram-negative bacterium (Group IV d) which was only later identified as* Ralstonia pickettii* [8]. The case was a 33-year-old African American male who had persistent positive blood cultures with a Group IV d Gram-negative bacillus resistant to all attempted antibiotics (ampicillin, penicillin G, and chloramphenicol). Autopsy was refused; however the patient was noted to have persistent positive blood cultures, a IV/VI harsh systolic murmur at the apex transmitting to the axilla, and fevers, suggesting endocarditis due to persistent bacteremia as cause of death [7].

More recent outbreaks of* Ralstonia pickettii* infections are documented as nosocomial outbreaks related to the use of contaminated medical solutions (saline, sterile water, disinfectants, intravenous ranitidine, and narcotics) used in patient care [9–17]. In Table 2 we provide a comprehensive review of literature to date from 2005 onward that reflects possible contamination sources as well as outcomes. Prior to 2006 Ryan et al. provide an excellent comprehensive review [1]. The presumptive ability of* Ralstonia* to persist in these sterile solutions is thought to be associated with its ability to survive within a wide range of temperatures (15°C–42°C) and pass through both 0.2 and 0.45 *μ*m filters, which are used to filter-sterilize medical solutions [18]. In a review of the literature there have been 55 cases of* Ralstonia* species infections, ranging from bacteremia to meningitis. The majority of infections reported have been treated with piperacillin, imipenem plus amikacin, and a combination of unnamed cephalosporins and aminoglycoside, as well as meropenem. There is no standardized recommendation for the treatment of* Ralstonia* infection because of the differences in sensitivities in particular to the carbapenems and aminoglycosides as well as the range of disease which includes asymptomatic to frank sepsis as in our patient. Only eight documented cases have resulted in death. The first case was the index case in 1968 as described above [7]. Two cases were elderly diabetic patients who died from complications of* R*.* pickettii* septicemia as a result of contaminated ion-exchange resins used to purify water for hospital use [19]. The ion-exchange resins used for deionization of city water allowed the survival of bacteria normally found in the city water supply while the bacteriological filters downstream only lowered the contamination level. Four premature infants have died from complications of* R. pickettii*-related infections. Of the four cases, one was pneumonia [20] and the other three were associated with bacteremia and sepsis [21, 22]. Finally, the eighth documented case resulting in death is our 51-year-old female who developed endocarditis due to complications of* Ralstonia pickettii* bacteremia with perivalvular abscess.

Immunocompromised patients seem to be at the highest risk of infection with pulmonary and blood stream infections being the primary routes [23]. Patients with acquired (i.e., HIV) or pharmaceutical (i.e., steroids, TNF blockers) induced immunosuppression are the most likely to succumb to infection with* Ralstonia* species. The single most important risk factor for acquiring infection with* R. pickettii* is cystic fibrosis. Furthermore, while respiratory tract and other nonsystemic infections responded well to parenteral antibiotic therapy, it seems to have little success in cases of* R. pickettii* bacteremia and sepsis, in particular, if a contaminated central venous line is involved. Removal of any indwelling device such as a central venous catheter is mandatory and critical in source control.

Interestingly, our patient had several predisposing risk factors that placed her at risk for both* R*.* pickettii* infection and complications. Approximately two months prior to presentation, our patient had a central venous catheter placed for intravenous iron transfusions. She also underwent tarsal tunnel release three weeks prior to presentation. In each of these settings she was exposed to not only potentially contaminated infusions but also hospital related procedures that may have resulted in infectious complications. Previous outbreaks have implicated hospital water, distilled water, saline, ion exchange resins, IV ranitidine, hemodialysis machines, and intravenous drug use [1]. Fortunately, our patient was an isolated case with no other cases suggesting that this was not a hospital associated outbreak. Finally, she was found to have a bicuspid aortic valve, which, in bacteremia, has been associated with increased incidence of infective endocarditis (IE) when compared to those without bicuspid aortic valves. Cases of IE occurring in patients with bicuspid aortic valves as compared to native valves have increased incidence of complications such as valve perforation, valve destruction, heart failure, and valvular, perivalvular, and/or myocardial abscess [24, 25].

Patients with health care-associated infections or who have had recent hospitalization or medical intervention (as in our case) are a new risk group that requires careful diagnostic attention in the presence of fever and bacteremia to evaluate infective endocarditis.* Ralstonia pickettii* should be considered an important potential etiology of nosocomial infections among patients who are immunocompromised, have cystic fibrosis, have central venous catheters, or have had recent surgical or medical hospitalizations. It is important to quickly recognize and treat* R. pickettii* as it has been identified as causing many potentially harmful infections resulting in increased morbidity and mortality.* Ralstonia* species are thought to be a rare infectious organism; however, our review of the literature suggests that the organism may be a more widespread and invasive pathogen than previously thought.

## Figures and Tables

**Table 1:** Blood culture results as referenced by days after hospital admission with day 1 being the day of admission. Surgical intervention with aortic valve replacement occurred on day 6 of hospitalization. Organism identified in all cultures was *Ralstonia pickettii*.

DPA	Culture source	Culture result	TTP hours
1	Blood	+	<24
2	Blood	+	<24
3	Blood	+	<24
4	Blood	+	<24
6	Aorta	+	<24
7	Blood	−	NA
8	Blood	−	NA
9	Blood	−	NA

DPA: days postadmission; TTP: time to positivity in hours.

**Table 2:** Comprehensive review of the literature from 2005 of cases of *Ralstonia pickettii* infection, traced source(s), and treatments where available.

Overview of *Ralstonia* species infections								
	*n*	Age (years)	Gender	Potential offending agent	Infection	Antibiotics used	Surgery	Outcome
Our case	1	51	Female	Iron infusions	Endocarditis	Levofloxacin TMP-SMX	AVR	Died

Graber et al. [7]	1	33	Male	IV drug abuse	Endocarditis^∗^	Penicillin + chloramphenicol	None	Died

Poty et al. [19]	4	Adults	—	Ion-exchange resin	Bacteremia	—	None	2 died 2 survived

Timm et al. [20]	10	Neonates	—	Acetic acid cleaning solution	Pneumonia	—	None	1 died 9 survived

Moreira et al. [21]	13	Adults	—	“Sterile” water for injection	Bacteremia	Ciprofloxacin + gentamicin	None	All survived
	3	Neonates	—		Bacteremia Pneumonia	Ampicillin + gentamicin	None	2 died 1 survived

Vitaliti et al. [22]	1	26 weeks gestation	Female	—	Bacteremia	Cephalosporin + meropenem + aminoglycoside	None	Died

Stelzmueller et al. [23]	38	Adults	—	None identified	Pneumonia Bacteremia	—	None	All survived

Forgie et al. [26]	2	Neonates	—	ECMO circuits	Bacteremia	Tobramycin + piperacillin-tazobactam	None	1 died 1 survived

Kismet et al. [27]	2	Toddlers	Females	Port-A-Cath	Bacteremia	Meropenem + cefepime	Removal of Port-A-Cath	All survived

Kimura et al. [28]	18	Neonates	—	Heparin flush	Bacteremia	Piperacillin	None	All survived

Strateva et al. [29]	1	75	Female	Hemodialysis system	Bacteremia	Levofloxacin	None	Survived

Fernández et al. [11]	46	—	—	IV Ranitidine	Bacteremia	—	None	All survived

Kahan et al. [30]	6	—	—	0.05% chlorhexidine	Bacteremia	—	—	—

Roberts et al. [10]	19	—	—	“Sterile” water	Bacteremia	—	—	—

Raveh et al. [31]	4	—	—	IV catheters	Bacteremia	—	—	—

Fujita et al. [32]	1	53	Male	IV catheter	Bacteremia	Cefazolin	None	Survived

Mikulska et al. [33]	10	—	—	None identified	Bacteremia	3rd generation cephalosporins + amikacin or carbapenems	None	1 died 9 survived

Woo et al. [34]	1	7	Male	Cord blood transplant	Bacteremia	Cefoperazone/sulbactam + ciprofloxacin	None	Survived

Marroni et al. [35]	9	Adults	—	Heparin solution	Bacteremia	—	None	All survived

Adiloğlu et al. [36]	1	Neonate	—	Distilled incubator water	Bacteremia	—	None	Survived

Candoni et al. [37]	20	Adults	—	None identified	Bacteremia	—	None	All survived

Japp et al. [38]	1	—	—	None identified	Bacteremia	—	None	—

Hansen et al. [39]	1	—	—	IV catheter	Bacteremia	—	None	Survived

Chomarat et al. [40]	1	—	—	None identified	Bacteremia	—	—	Survived

Lazarus et al. [41]	1	Adult	—	In vitro handling process	Bacteremia	—	None	Survived

Marroni et al. [35]	6	—	—	Purified saline	Bacteremia	—	None	All survived

Yoneyama et al. [42]	17	Adults	—	0.05% chlorhexidine aqueous solution	Bacteremia	—	None	All survived

Lacey and Want [9]	7	Children	—	“Sterile” distilled water	Bacteremia	—	None	All survived

Chetoui et al. [43]	6	Adults	—	“Sterile” saline	Bacteremia	—	None	All survived

Maki et al. [12]	9	Adults	—	Pre-drawn fentanyl syringes	Bacteremia	—	None	All survived

Gardner and Shulman [13]	9	Infants	—	Tracheal irrigant solution	Respiratory infection	—	None	All survived

Trotter et al. [44]	1	5	Male	None identified	Pneumonia	TMP-SMX	Decortication	Survived

Hagadorn et al. [45]	1	Neonate	—	Home water birth	Pneumonia	—	—	—

Miñambres et al. [46]	1	Adult	—	None identified	Pneumonia	—	None	Survived

MMWR [16]	13	Children	9 Female 4 Male	0.9% sodium chloride solution	Colonization respiratory infection	—	None	All survived

MMWR [14]	5	Infants	—	0.9% sodium chloride solution	Colonization respiratory infection	—	None	All survived

Labarca et al. [17]	34	—	—	0.9% sodium Chloride	Pneumonia bacteremia	—	None	5 died 29 survived

Pan et al. [47]	1	65	Male	None identified	Pneumonia	Imipenem-cilastatin	Chest tube	Survived

Ahkee et al. [48]	1	41	Male	Respiratory therapy solution	Pneumonia	Aztreonam piperacillin	Thoracentesis	Survived

Kendirli et al. [49]	2	2 months	Female	Ventilator circuit	Pneumonia bacteremia	Piperacillin-tazobactam	None	Survived
		14	Male		Pneumonia bacteremia		None	Died

Burns et al. [50]	2	Adults	—	Cystic fibrosis	Respiratory infection	—	None	All survived

Wertheim and Markovitz [51]	1	71	Male	None identified	Osteomyelitis	TMP-SMX	Laminectomy	Survived

Degeorges et al. [52]	1	29	Male	None identified	Osteomyelitis	—	Debridement	Survived

Elsner et al. [53]	1	Adult	—	Hemodialysis machine	Spinal osteitis	—	—	—

Sudo et al. [54]	1	48	Female	None identified	Spondylitis	Cefepime + minocycline	None	Survived

Zellweger et al. [55]	1	—	Male	IV drug abuse	Septic arthritis	Ceftriaxone	—	Died

Makaritsis et al. [56]	1	83	Female	None identified	Septic arthritis	Ceftazidime	Arthrocentesis	Survived

Heagney [57]	1	—	—	None identified	Meningitis	—	—	—

T'Sjoen et al. [58]	1	38	Female	Ventriculoarterial shunt	Meningitis	—	—	—

Fass and Barnishan [59]	1	—	—	None identified	Meningitis	—	None	—

Yuen et al. [60]	1	32	Male	None identified	Peritonitis	Cefuroxime	Paracentesis	Survived

Carrell et al. [61]	1	—	Male	—	Seminal infection	—	None	Survived

Parent and Mitchell [62]	8	Adults	—	Crohn's disease	Infection	—	None	All survived

Minah et al. [63]	—	—	—	Myelosuppressed cancer	Asymptomatic	—	—	Survived

McNeil et al. [15]	5	Infants	—	Respiratory therapy solution	Asymptomatic	—	None	All survived

Costas et al. [64]	28	Infants	—	None identified	Pseudooutbreak	—	None	All survived

Heard et al. [65]	15	Infants	—	Contaminated bottles	Pseudooutbreak	—	None	All survived

Dimech et al. [8]	6	Adults	—	Detergent disinfectant	Pseudobacteremia	—	None	All survived

Lacey and Want [9]	25	Adults	—	Blood culture technique	Pseudobacteremia	—	None	All survived

Boutros et al. [66]	14	—	—	Culture bottles	Pseudobacteremia	None	None	All survived

Maroye et al. [67]	6	Children	—	Distilled water	Asymptomatic	—	None	All survived

Morar et al. [68]	1	Child	—	None identified	Asymptomatic	—	None	Survived

Yoneyama et al. [42]	7	—	—	Water	Asymptomatic	—	None	Survived

ECMO: extracorporeal membrane oxygenation; IV: intravenous; AVR: aortic valve replacement; TMP-SMX: trimethoprim-sulfamethoxazole.

^∗^Presumed to have endocarditis but could not confirm diagnosis.
